# Applicability of selected Brighton Collaboration case definitions in low-resource settings: A prospective hospital-based active surveillance in Addis Ababa, Ethiopia

**DOI:** 10.1016/j.vaccine.2024.06.046

**Published:** 2024-06-21

**Authors:** Kassech Sintayehu, Anna Shaum, Zelalem Tazu Bonger, Eden Dagnachew Zeleke, Biniyam Tedla Mamo, Abenezer Abraham Anito, Dawit Bekele, Ashley T. Longley, Habtamu Gashaw, Asnakech Alemu, Desalegne Degefaw, Shu-Hua Wang, Wan-Ting Huang

**Affiliations:** a Ohio State Global One Health, Addis Ababa, Ethiopia; b Global Immunization Division, Centers for Disease Control and Prevention (CDC), Atlanta, GA, USA; c Ethiopian Food and Drug Authority (EFDA), Addis Ababa, Ethiopia; d Ohio State University Global One Health Initiative (OSU GOHi), Columbus, OH, USA; e Brighton Collaboration, Task Force for Global Health, Decatur, GA, USA; f Global Health Program, College of Public Health, National Taiwan University, Taipei, Taiwan; g National Taiwan University Children’s Hospital, Taipei, Taiwan

**Keywords:** Brighton Collaboration, Case definitions, Active surveillance, Vaccine safety

## Abstract

**Introduction:**

Standardizing case definitions for priority vaccine safety conditions facilitates uniform evaluation and consolidation of data obtained from different settings. The Brighton Collaboration case definitions (BCCD) were created to support this harmonization and enable classification from level 1 (most certain) to level 5 (not a case) of certainty. Assessing the performance of BCCD in practice is critical, particularly in resource-limited settings, where many new vaccines may be introduced without prior monitoring in high-income countries. We assessed the performance of BCCD in Addis Ababa, Ethiopia, as applicable to COVID-19 and other vaccines.

**Methods:**

Active surveillance was conducted at Tikur Anbessa Specialized Hospital, the largest referral hospital in Ethiopia. During June 1, 2022–May 31, 2023, three trained physicians prospectively identified patients eligible for COVID-19 vaccination (regardless of vaccine receipt) who presented with one or more of eleven pre-specified adverse events of special interest (AESI) from the emergency department and inpatient wards. Standardized data collection forms were used to capture patient information and assign level of certainty (LOC), regardless of vaccination status for COVID-19. We conducted descriptive analysis to characterize cases and the LOCs reached for each AESI.

**Results:**

We detected 203 AESI cases. The most detected conditions were thrombosis and thromboembolism (n = 100, 49 %) and generalized convulsions (n = 38, 19 %). Ninety-six percent of the cases were confirmed at levels 1–3 (n = 187) or level 5 (n = 9) LOC. Non-classifiable (level 4) cases were observed for pericarditis (n = 2), encephalitis (n = 2), myelitis (n = 2), and generalized convulsion (n = 1).

**Conclusion:**

The BCCD were successfully applied in > 95 % of cases in a large referral hospital in Ethiopia, with generalized convulsion, pericarditis, and encephalomyelitis as the exceptions. We recommend further evaluation in other low-resource settings, particularly in rural or non-referral hospitals, to gain additional insights into performance of these definitions for revision or adaptation, as needed.

## 1. Introduction

Every vaccine is evaluated for safety, immunogenicity, and efficacy in several phases of clinical trials before it is approved for use. Clinical trials typically enroll between a few hundred to ten thousand participants and have limited duration of follow-up, and thus are inadequately powered to identify rare adverse events or longer-term safety concerns. This is where post-marketing surveillance is key to understanding a vaccine’s safety following licensure [1]. A critical component of the scientific evaluation of adverse events following immunization (AEFI) across the vaccine lifecycle is a standardized and shared vocabulary to allow for the comparability of data [2,3]. Adverse events of special interest (AESIs) are predefined medically significant events that have the potential to be causally associated with a vaccine product and that need to be carefully monitored and confirmed by further specific studies [5,6]. Rare but serious AEFIs are easily missed or misclassified without standardized case definitions; the use of standardized case definitions, therefore, enables harmonized assessment and aggregation of data collected across clinical trials, epidemiologic studies, and AEFI surveillance systems, both in resource-limited as well as resource-rich settings [5].

The Brighton Collaboration was founded in 2001 to facilitate the development, evaluation, and dissemination of high-quality information about the safety of human vaccines. Standardized case definitions developed by the Brighton Collaboration include criteria for the categorization of clinical events with levels of diagnostic certainty (LOC) to measure the specificity of an AEFI: level 1 (most certain with the highest level of specificity), level 2 (intermediate specificity and more sensitive for the respective AEFI), level 3 (lowest specificity and highly sensitive for the respective AEFI), level 4 (insufficient information to confirm a case), and level 5 (not a case). This classification accounts for the availability of personnel, clinical, and laboratory diagnostic facilities and aims to allow consistent comparability in diverse settings with varying availability of clinical resources. As of October 2023, case definitions for more than 70 conditions were available. Although the Brighton Collaboration case definitions (BCCD) were to be reviewed for applicability, reliability, sensitivity, and specificity in their intended settings of use, this has mostly not occurred [4]. Assessing the field performance of these definitions is therefore critical, especially in resource-poor settings where many new vaccines (e.g., RTS,S [malaria vaccine], typhoid conjugate vaccines, novel oral polio vaccine type 2) used in these settings may be introduced without prior monitoring in high-income countries.

Since 2022, Ethiopia has implemented a prospective hospital-based sentinel surveillance for selected AESIs at Tikur Anbessa Specialized Hospital (TASH) to monitor the safety of newly introduced COVID-19 vaccines. In this assessment, we reviewed reports of AESIs collected at this surveillance site to describe the utility and applicability of BCCD. The specific objectives were to (1) describe the LOC for AESI cases assessed at TASH; (2) describe reasons for being unable to classify cases (level 4); and (3) assess completeness of each BCCD criterion, specifically for Guillain-Barré syndrome (GBS), given its association with adenovirus vector COVID-19 vaccines [7] and the complexity of the GBS BCCD compared to other conditions.

## 2. Methods

### 2.1. Surveillance setting and population

The surveillance was conducted at TASH, Addis Ababa University, the largest public referral and teaching hospital in Ethiopia and located in the capital city, Addis Ababa. It is also an institution where many specialized clinical services that are not available in other public or private institutions are rendered to patients coming from all over the country. [8].

In April 2022, TASH implemented AESI case surveillance to prospectively identify prespecified conditions among patients eligible for COVID-19 vaccination (as per the standard of the national immunization program), who presented to the emergency outpatient department (EOPD) or who were hospitalized. The pilot period ended on May 31 and formal data collection started in June. For this analysis we included data collected from June 1, 2022 to May 31, 2023.

### 2.2. Case definitions evaluated

The Brighton Collaboration Safety Platform for Emergency vACcines (SPEAC) project is funded by the Coalition for Epidemic Preparedness Innovations to facilitate harmonized safety assessment of novel vaccines for COVID-19 and other priority pathogens with epidemic or pandemic potential. SPEAC has identified a list of AESI relevant to COVID-19 vaccines [9], prioritized and made available standardized case definitions for these AESIs, and created companion guides with structured data collection forms. Additionally, SPEAC developed algorithms (case logic and pictorial decision trees) to support LOC determination based on the information collected [10,11]. Eleven AESIs were chosen by Ethiopian Food and Drug Authority (EFDA) for prospective surveillance: myocarditis/pericarditis, abscess at injection site, thrombosis with thrombocytopenia syndrome (TTS), thrombocytopenia, thrombosis and thromboembolism, anaphylaxis, acute disseminated encephalomyelitis (ADEM), Bell’s palsy, generalized convulsion, GBS and Fisher syndrome, and encephalitis/myelitis.

### 2.3. Data collection

Three trained officers (medical doctors) reviewed patient log sheets and medical records at the beginning of each workday and used reported symptoms consistent with the AESIs or provisional diagnoses to search for potential cases from EOPD (excluding non-medical cases such as trauma, accident, poisoning, or mental illness) and inpatient wards. Whenever an AESI was identified, the officers obtained consent from the patient and reviewed individual patient charts to extract relevant clinical information and other demographic as well as COVID-19 vaccination details, when applicable (for subsequent analyses). Structured data collection forms were developed or adapted from the SPEAC case definition companion guides [9] to capture patient information, including the key criteria of Brighton Collaboration LOC ascertainment.

Abstracted data were recorded on paper data collection forms by surveillance officers who then manually applied the Boolean logic interpretation tables or pictorial algorithms in the companion guides to determine the LOC for each AESI, regardless of vaccination status for COVID-19 [12]. The completed paper forms were then entered into a secured Open Data Kit (ODK) [13] database specifically designed for this project, by a trained data clerk, ensuring the anonymity of the data. To validate the accuracy of the data collection, case reviews were done with a consultant (W-TH). The ODK forms also integrated Boolean logic for automatic determination of the LOC; any discrepancies between the manual and automatic LOC classification were reviewed and verified by the data clerk during data entry and by the data manager and other project staff during regular site monitoring visits.

### 2.4. Data analysis

Descriptive analysis was performed to characterize patient demographics and the LOCs for each AESI. We calculated the proportion of all cases that met each LOC and described reasons for cases being classified as level 4 (non-classifiable due to insufficient information). Last, we assessed the prevalence of each BCCD criterion for GBS.

### 2.5 {}. Ethical approval

The protocol was approved by institutional review board of the Ethiopian Public Health Institute and Addis Ababa University College of Health Science, with reference numbers EPHI-IRB-408–2021 and 009/22/SoP respectively. The U.S. Centers for Disease Control and Prevention reviewed the protocol, and it was deemed non-research, program implementation.

## 3. Results

Overall, 203 AESI cases were detected at TASH (Table 1); 162 (80 %) detected from EOPD. Among all patients with AESIs, females slightly predominated (n = 119, 59 %), as well as those with residence in Addis Ababa (n = 117, 58 %). The most frequently detected cases were thrombosis and thromboembolism (n = 100, 49 %), generalized convulsion (n = 38, 19 %), and thrombocytopenia (n = 23, 11 %); the median age was higher for patients with thrombosis and thromboembolism (49 years, range 16–88) than patients with other AESIs. No cases of abscess at injection site, TTS, or anaphylaxis were identified.

**Fig. 1.**
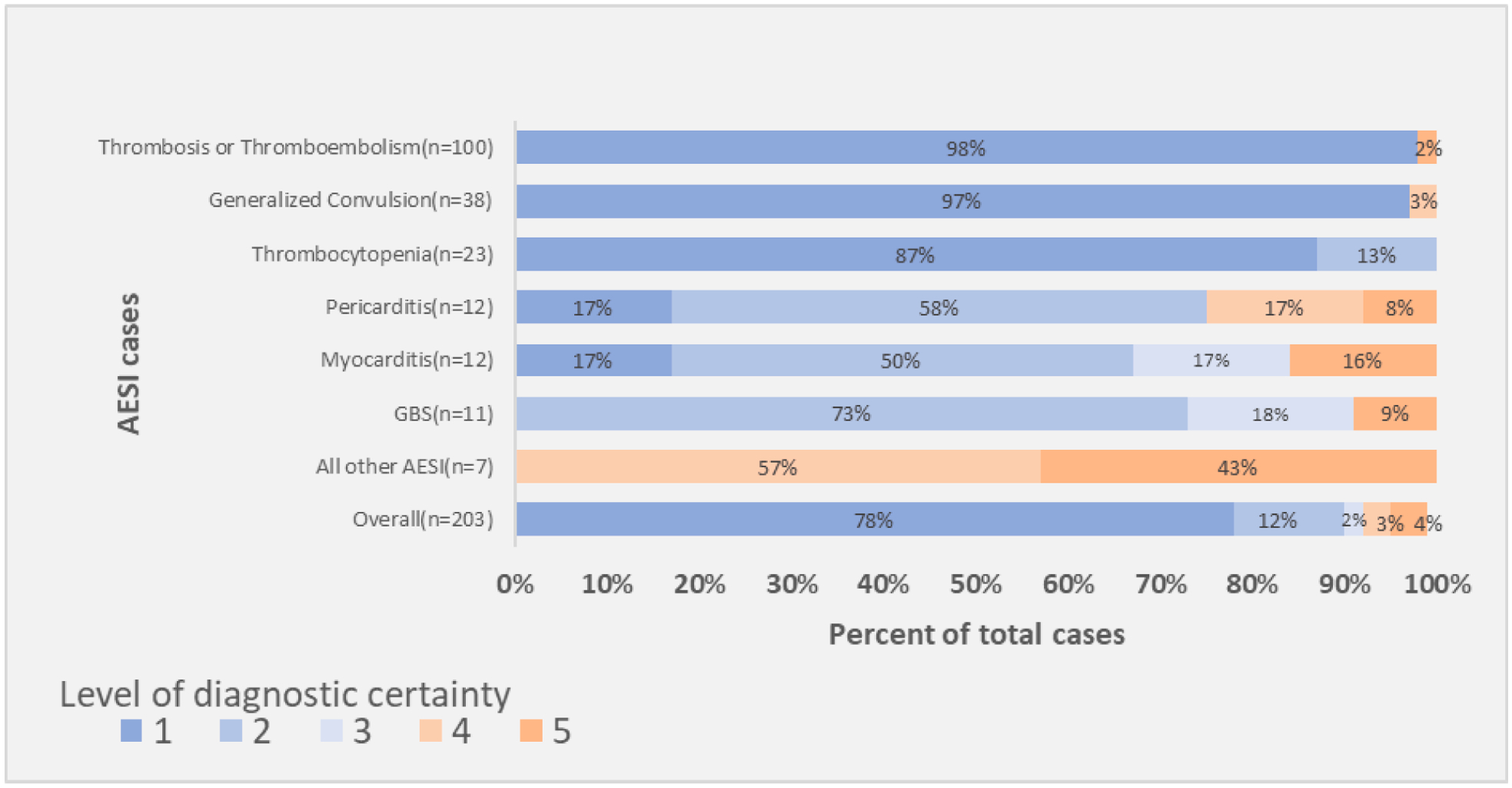
The Brighton Collaboration level of diagnostic certainty for AESI cases, Tikur Anbesa Specialized Hospital, Ethiopia, June 2022–May 2023 (n = 203) AESI, adverse event of special interest; ADEM, acute disseminated encephalomyelitis. Level 1: Highest specificity of a case Level 2: Intermediate specificity of a case, higher sensitivity Level 3: Lowest specificity of a case, highest sensitivity Level 4: Insufficient information to confirm a case Level 5: Not a case All other AESI (n = 7) included four level 4 (2 encephalitis and 2 myelitis) and three level 5 (2 ADEM and 1 Bell’s palsy) cases.

Overall, 187/203 (92 %) AESI cases were confirmed at levels 1–3 LOC. This included all (100 %) thrombocytopenia cases, 98 % of thrombosis and thromboembolism cases, 97 % of generalized convulsion cases, 91 % of GBS cases, 84 % of myocarditis cases, and 75 % of pericarditis cases (Fig. 1). All the other AESI cases were either non-classifiable (level 4, n = 4 [2 %]) or not a case (level 5, n = 3 [2 %]).

Non-classifiable cases among all AESIs included generalized convulsion (n = 1) because it was unknown whether the movements during the seizure involved both right and left limbs, pericarditis (n = 2) because of unknown chest radiograph and electrocardiogram findings, and encephalitis (n = 2) and myelitis (n = 2) because there was no data on indicators of central nervous system inflammation.

Table 2 summarizes the prevalence of each criterion in the BCCD for the GBS cases, given their association with COVID-19 vaccines and the complexity of the GBS case definition. Bilateral weakness of limbs and characteristic temporal illness pattern were the most prevalent criteria documented, occurring in 100 % of GBS cases. Data on electrophysiologic studies were available in 8 (73 %) cases; of these, 7 (88 %) had findings consistent with GBS. The cytoalbuminologic dissociation criterion, including cerebrospinal fluid (CSF) total white cell count <50 cells/uL, with or without CSF protein elevation, however, was unknown in the majority (n = 10, 91 %) of the cases. The most common phenotype of GBS was acute motor axonal neuropathy (AMAN) (n = 4), per nerve conduction studies and electromyogram.

## 4. Discussion

We aimed to evaluate the applicability of the BCCD for selected AESIs in the context of a prospective data collection process at TASH — the largest hospital in Ethiopia. Our findings suggest that most of these BCCD were applicable in a resource-limited country like Ethiopia, with generalized convulsion, pericarditis, and encephalomyelitis as the exception. Validation of the reported AEFI is essential before proceeding with causality assessment by national or sub-national committees. Further, the WHO recommends adoption of the BCCD to validate the AEFI diagnosis, if available, for use in causality assessment [14]. The results could also be used as a foundation for further studies applying BCCDs in low- and middle-income countries (LMIC) or monitoring vaccine safety after the introduction of new vaccines.

All the GBS cases identified at TASH were classifiable (meeting levels 1–3 or 5 LOC), but none of them achieved the highest level of certainty; the majority were categorized as level 2 LOC. In addition to clinical criteria, a level 2 LOC requires either relative absence of CSF pleocytosis (<50 WBC/uL) or electrophysiological patterns consistent with GBS, and to further reach level 1 LOC, both cytoalbuminologic dissociation in CSF and acute peripheral neuropathy criteria are required [15]. While 88 % of the tested patients had typical GBS electrophysiological features (mostly AMAN), cytoalbuminologic dissociation was observed in only one GBS case for which CSF analysis was done. In our setting, drivers of a particular testing decision in clinical practice include local guideline standards, out-of-pocket cost of tests, and whether utilization of such tests can inform subsequent patient treatment. In observational settings, variations in test ordering, particularly non- or under-testing, could affect the interpretation on the applicability of the GBS case definition because key data elements are not available. In our surveillance, the presence of CSF results was comparable to those from a Korean cohort (23 %) [15,16], but lower than those reported in Germany (75 %–80 %) [17], Malaysia (73 %) [17], and Bangladesh (89 %) [19]. Also, in contrast to our cases of predominantly AMAN subtype, GBS has been mostly shown to be the demyelinating subtype in Ethiopia [19,20], North America and Europe [17,15].

Mild myocarditis has been observed following receipt of mRNA COVID-19 vaccines [21]. In the BCCD for myocarditis, the level 1 LOC was reached either by histopathologic demonstration of myocardial inflammation, or by a combination of elevated myocardial biomarkers with an abnormal cardiac magnetic imaging (cMR) or echocardiography [22]. In Ethiopia, endomyocardial biopsy and cMR are less available because these tests are limited only to some facilities; myocardial biomarkers are also not always conducted at TASH. Despite all this, however, 17 % of the myocarditis cases identified in our surveillance were classified as level 1 LOC; this proportion, nevertheless, was lower as compared to findings from studies in Australia (35 % as level 1 LOC)[23] and Sweden (39 %–42 % as level 1 LOC) [19], but comparable with results from a retrospective review of spontaneous AEFI reports retrieved from the U.S. Vaccine Adverse Event Reporting System (13 % as level 1 LOC) [18].

While a small number of pericarditis and encephalomyelitis cases characterized the majority of our level 4 LOC cases (unclassifiable), this was due to the lack of test results for these conditions. Our two level 4 LOC pericarditis cases lacked chest radiograph or electrocardiogram results to achieve at least level 3 LOC, but these tests are not specialized and are routinely available. For the encephalitis and/or myelitis cases that met level 4 LOC, we lacked information on nonspecific (fever) or neurodiagnostic (CSF pleocytosis, electroencephalography, or neuroimaging) CNS inflammatory indicators; nevertheless, the overall number of cases was low.

We detected most AESI cases at the EOPD because most patients admitted to the inpatient wards at TASH enter through the EOPD, where they may stay for days, as inpatient beds are not readily available. Additionally, 42 % of our patients lived outside of Addis Ababa, highlighting the role of TASH as a referral hospital for the country. This makes calculating background rates using population denominators challenging, as TASH treats patients from across Ethiopia, and not a specific catchment population in Addis Ababa. Future work could focus on generating site (hospital) specific background rates for new vaccine introductions in the absence of a true denominator for the catchment population.

In our surveillance, data collectors received training on the tools used to apply BCCD. Instead of having two collectors review the same AESI case to ensure the reliability of LOC determination, we compared manual and automatic ODK LOC at data entry and site monitoring visits to assure LOCs are consistently applied. The value of each BCCD criterion and LOC automatically generated by ODK was further verified by the consultant. Whenever there was a revision of the BCCD (e.g., anaphylaxis [23]) or a change on the LOC algorithm (e.g., GBS), the ODK forms were updated accordingly, and data already collected were retrospectively applied against the updated components and Boolean logic to determine LOC. For efficiency and accuracy, SPEAC is transforming the structured case definition formats and algorithms in the companion guides into a validated online Automatic Brighton Classification (ABC) tool. This ABC tool would allow users to automatically assess the LOC for a BCCD based on data inputs [25,26].

Our study has limitations. The accuracy and availability of information relied on the data quality in the medical records. Missing data and lack of clarity on documented clinical notes led to difficulties assigning LOC. Additionally, standard coding of diagnosis was not widely used at TASH; screening and identification of potential cases depended on the surveillance officers’ interpretation of the main symptoms and diagnoses from the patient log sheets. We could have missed cases that presented with non-AESI conditions or with atypical presentation of AESI for which subsequent laboratory investigations found abnormal laboratory test results (e.g., thrombocytopenia) consistent with an AESI.

In conclusion, BCCD was applicable in Ethiopia with few constraints. This is one of the few evaluations of existing and new BCCDs for AESIs relevant to COVID-19 vaccines in the African region. Prior to the COVID-19 pandemic, only selected case definitions have been utilized and evaluated in low-resource settings [24]. The paper and ODK data collection forms created can be used as templates for application of the BCCD in vaccine safety and other epidemiologic studies globally. The implementation of BCCD facilitates generation of standardized vaccine safety data from TASH and will contribute to global data on vaccines. Further evaluation in similar settings, particularly non-referral or rural hospitals in LMICs, could provide additional insights aimed at revision or adaptation of the BCCD. Furthermore, training of healthcare professionals on how to best implement these case definitions and the inclusion of LMIC experts into the BCCD working groups can help address potential impediments to the application of case definitions in LMICs.

## Figures and Tables

**Table 1:** Characteristics of patients with AESIs at Tikur Anbesa Specialized Hospital, Ethiopia, June 2022–May 2023.

Condition	Detected at EOPD, n (%)^1^	Detected at IPD, n (%)^1^	Detected at EOPD and IPD, n (%)^1^	Median age (range)^2^, years	Male, n (%)^2^	Living in Addis Ababa, n (%)^2^
Total AESIs	162 (100)	41 (100)	203 (100)	38 (13, 88)	84(41)	117 (58)
ADEM	1 (<1)	1 (2)	2 (<1)	24 (21, 26)	0 (0)	2 (100)
Bell’s palsy	1 (<1)	0 (0)	1 (<1)	52 (52, 52)	1 (100)	0 (0)
Encephalitis	1 (<1)	1 (2)	2 (<1)	24 (21, 26)	0 (0)	2 (100)
GBS	7 (4)	4 (10)	11 (5)	26 (15, 58)	4 (36)	6 (55)
Generalized convulsion	35 (22)	3 (7)	38 (19)	31 (14, 82)	22 (58)	34 (90)
Myelitis	1 (<1)	1 (2)	2 (<1)	24 (21, 26)	0 (0)	2 (100)
Myocarditis	1 (<1)	11 (27)	12 (6)	29 (17, 83)	8 (67)	6 (50)
Pericarditis	1 (<1)	11 (27)	12 (6)	29 (17, 83)	8 (67)	6 (50)
Thrombocytopenia	21 (13)	2 (5)	23 (11)	35 (13, 62)	9 (39)	13 (57)
Thrombosis and thromboembolism	93 (57)	7 (17)	100 (49)	49 (16, 88)	32 (32)	46 (46)

AESI, adverse event of special interest; ADEM, acute disseminated encephalomyelitis; GBS, Guillain-Barré syndrome; EOPD, emergency outpatient department; IPD, inpatient department.

1 Values are column %.

2 Values are % of total cases detected at EOPD and IPD for that AESI.

**Table 2:** Prevalence of each case definition criterion in Guillain-Barré syndrome patients, Tikur Anbesa Specialized Hospital, Ethiopia, June 2022–May 2023.

Criterion category	(n = 11)^1^
Weakness of limbs	
Bilateral weakness of limbs	11/11(100)
Decreased or absent DTRs	
Decreased or absent DTRs in weak limbs	10/11 (91)
Characteristic temporal illness pattern	
Time between onset-nadir 12 h to 28 days	11/11 (100)
Electrophysiologic studies consistent with GBS	
AIDP, AMAN and/or AMSAN	7/8 (88)
Cytoalbuminologic dissociation in CSF	
CSF WBC < 50 cells/uL with CSF protein elevation	1/1 (100)
CSF WBC < 50 cells/uL without CSF protein elevation	0/1 (0)
Alternative diagnosis	
Absence of identified alternative diagnosis for weakness	10/11 (91)

DTR, deep tendon reflex; AIDP, acute inflammatory demyelinating polyneuropathy; AMAN, acute motor axonal neuropathy; AMSAN, acute motor and sensory axonal neuropathy; CSF, cerebrospinal fluid; WBC, total white cell count.

1 Values are n/N (%), where n is the case count of that row and N is the number of cases with data available for that row.

## Data Availability

The authors do not have permission to share data.
